# Associations between body mass index and pubertal development based on the outcomes of girls with early breast development

**DOI:** 10.3389/fendo.2022.991908

**Published:** 2022-10-21

**Authors:** Manman Zhao, Meijuan Liu, Bingyan Cao, Chunxiu Gong

**Affiliations:** ^1^ Department of Endocrinology, Genetics and Metabolism, National Center for Children’s Health, Beijing Children’s Hospital, Capital Medical University, Beijing, China; ^2^ Department of Pediatrics, Beijing Luhe Hospital, Capital Medical University, Beijing, China

**Keywords:** premature thelarche, breast development recurrence, central precocious puberty, body mass index, luteinizing hormone

## Abstract

**Objective:**

To investigate the associations between physical and pubertal development based on the breast development outcomes in girls.

**Methods:**

This was a retrospective study. A total of 452 girls aged 6~8 years were included. Based on their breast development outcomes, the patients were divided into an idiopathic central precocious puberty (ICPP) group and a premature thelarche (PT) group. Anthropometry included measurements of height, weight, and BMI. ICPP was diagnosed when five diagnostic criteria from the current guidelines were met.

**Results:**

The girls with breast development at initial evaluation had a median age of 6.9 years. In total, 31.4% of patients were diagnosed with ICPP, and ICPP was rare in girls <7 years old (19%). Patients who presented with recurrence of breast development in the PT group accounted for 38.4%. At initial evaluation, the height, weight, BMI and bone age (BA) of the girls in the PT group corresponded to those of a normally developing girl at ages 7.8 years, 8.2 years, 8.6 years and 7.6 years, respectively. The girls in the ICPP group had a mean age of 7.3 years, and their mean height, weight, and BMI were 129.6 cm, 28.4 kg, and 17.0 kg/m^2^, which corresponded to the mean of a normally developing girl at ages 8.5 years, 9.1 years, and 10.5 years, respectively; these patients had BA of 9.2 years. Additionally, based on receiver operating characteristic (ROC) curve analysis, when the basal luteinizing hormone (LH) level was 0.32 IU/L and BMI reached 16.4 kg/m^2^, CPP was diagnosed in patients meeting all five diagnostic criteria for CPP, and the specificity and sensitivity were 71.9% and 88.2%, respectively.

**Conclusions:**

Girls with breast development before 8 years old had a high proportion of recurrence of breast development. The physical development of these girls at the time of breast development preceded that of normally developing girls by 1-2 years. BMI is an independent risk factor for early pubertal development, and is a simple and clear predictor of ICPP. In addition to the five classic diagnostic criteria, CPP is diagnosed when physical development corresponds to the mean for a 10.5 years old girl.

## Introduction

Female precocious puberty is defined as the development of secondary sexual characteristics before the age of 8 years (1). It is divided into central precocious puberty (CPP), peripheral precocious puberty (PPP), and partial forms of precocious puberty. CPP is initiated by the hypothalamic-pituitary-gonadal (HPG) axis, whereas PPP is not. Premature thelarche (PT) is partial precocious puberty without other accompanying manifestations of sexual maturation (2); it is also known as variant precocious puberty and is benign, requiring no intervention. Our group assessed female infantile patients under the age of 2 years with isolated early breast development and clinically followed up these patients for 5 years. Overall, 96.97% of them had a benign outcome (3). However, the occurrence of PT in atypical mini-puberty has been increasingly reported in the past 20 years, and the prevalence of PT has been reported to range from 2.2% to 4.7% among girls (4–6). This situation can be easily misdiagnosed as CPP. Studies have documented that patients benefit from gonadotropin-releasing hormone agonist (GnRHa) treatment-induced height gain only if the treatment is initiated before age 6; conflicting results have been reported when the treatment is initiated in patients at age 6-8 years (7–10).

Body mass index (BMI) is a practical, available, and widely used method to identify overweight and obesity in a population. Recent evidence has suggested a relationship between childhood obesity and the timing of pubertal milestones in girls (11, 12). However, whether early puberty is the cause or the consequence of increased body fat remains unclear.

In this study, we ruled out tumors or other anatomical abnormalities and then investigated the clinical data of girls aged 6-8 years who had breast development and visited our hospital’s outpatient department; these patients were followed up to determine the outcomes, such as the final diagnosis and physical development measurements of the children. In addition, physical and chemical biomarkers for diagnosing CPP were studied to help provide better diagnosis and treatment.

## Materials and methods

### Subjects and group stratification

This is a retrospective study. In all, 497 female children aged 6-8 years who visited the outpatient clinic of Beijing Children’s Hospital from January 2017 to October 2021 with “breast development” as the chief complaint were enrolled in this study, and clinical data and follow-up progress were assessed. Girls with “breast development” need to have well-established breast ultrasound, pelvic ultrasound, bone age, and related blood tests such as sex hormones and thyroid hormones, all of which are not possible to complete at one time and require an appointment, so we defined the initial evaluation as the completion of all required examination within one month. The children were categorized into a < 7-year-old group and a ≥ 7-year-old group based on their age at initial evaluation. Based on the follow-up outcomes, they were categorized into an idiopathic central precocious puberty (ICPP) group and a premature thelarche (PT) group. Written informed consent was obtained from a parent or guardian on behalf of each child and the study was approved by the ethics committee of Beijing Children’s Hospital, Capital Medical University. The two outcome groups diagnostic criteria were as follows.

### Diagnostic criteria

The CPP diagnostic criteria (1) (2015 Chinese guidelines) were as follows: (1) presence of secondary sexual characteristics before 8 years of age in girls; (2) linear growth acceleration (> 6 cm/yr); (3) advanced bone age (BA) (≥ 1 year); (4) uterine length diameter exceeding 3.4 cm, ovarian volume > 1 ml, and multiple follicles > 4 mm in diameter; and (5) gonadal axis function initiation: basal levels of luteinizing hormone (LH) > 0.83IU/L or a positive GnRH provocation test (peak LH ≥ 5IU/L and peak LH/peak follicle stimulating hormone (FSH) ≥ 0.6).

Suspected CPP was defined as patients who met the following combinations of the above CPP criteria: (1), (2) and (3) or (1), (2), (3) and (4). These patients were followed up to 8 years of age, and if they eventually met all five diagnostic criteria, they were diagnosed with CPP.

The ICPP judgment criteria were as follows: patients who fulfilled the diagnostic criteria for CPP, excluding those with the secondary causes.

The criteria for PT were as follows: the girl showed isolated breast development before the age of 8 years, without other signs of pubertal development during follow-up, such as linear growth acceleration and advanced BA; uterine length was less than 3.4 cm, and the ovarian volume was less than 1 ml; and unelevated basal levels of LH (less than 0.83IU/L) or a negative GnRH provocation test.

### Observations

All the subjects were interviewed in detail about their medical history and then underwent a physical examination that included measurements of height, weight, and calculation of BMI. Body weight and height were measured with the patient barefoot and wearing light clothes. BMI was calculated using the formula [BMI (kg/m^2^) =weight (kg)/height^2^ (m^2^)]. Breast development was determined according to Tanner staging and monitored every 3 months up to age 8 years. Serum sex hormone levels, including those of FSH, LH, estradiol (E2), and progesterone, were measured by immunochemiluminescence every 3 months. Pelvic ultrasound was performed every 3 months by professionally trained endocrine sonographer. BA was assessed using the G-P method every 6 months by the same pediatric endocrinologist in all times. Magnetic resonance imaging (MRI) of the pituitary or sellar region was performed in 332 patients. Those who did not undergo MRI had no signs or abnormal neurological findings indicating CNS tumors, during follow-up.

### Statistical methods

The data were processed and analyzed by SPSS 24.0 software. Normally distributed measurement data were expressed as the mean ± standard deviation (SD), and an independent samples test was used to compare the means between groups. Nonnormally distributed continuous variables were expressed as the median (25%, 75% quartiles), and the Mann–Whitney U test was used to compare the means between groups. The counting data were expressed as percentages. *P*<0.05 was considered statistically significant. Factors influencing the outcome of precocious puberty were analyzed by multivariate logistic regression analysis. A receiver operating characteristic (ROC) curve analysis was performed, and the area under the ROC curve (AUC) was calculated to evaluate the diagnostic value. The optimal cutoff value, sensitivity, and specificity were determined by calculating the Youden index.

## Results

**Figure 1:**
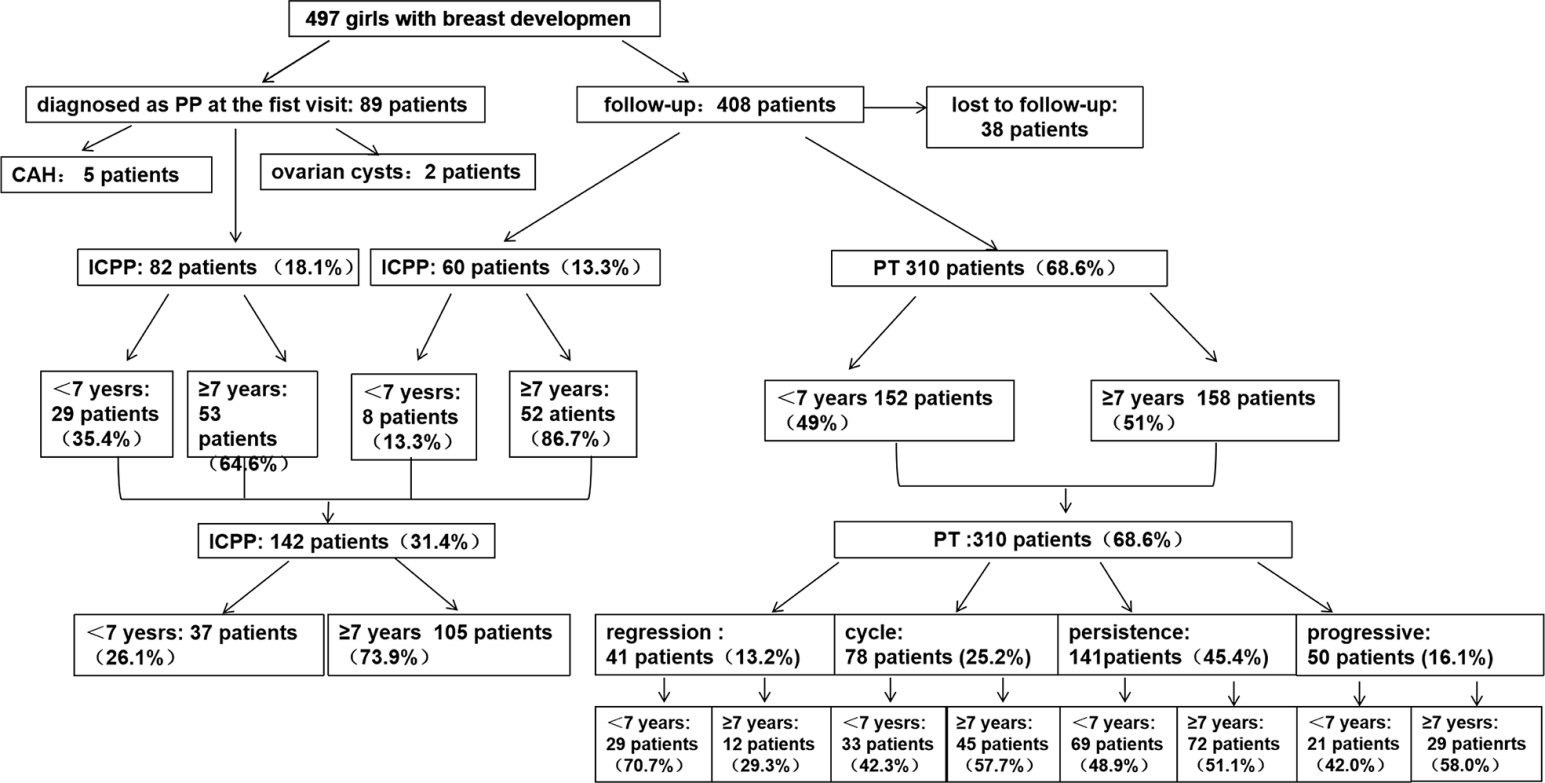
Flowchart of participant screening and enrollment. CAH, congenital adrenal hyperplasia; ICPP, idiopathic central precocious puberty; PT, premature thelarche.

A total of 497 girls were included at baseline, 38 were lost to follow-up, and 7 were excluded because of the following diseases: 5 with congenital adrenal hyperplasia (CAH) and 2 with ovarian cysts. Thus, 452 participants with follow-up data were analyzed. The group at baseline was divided, according to age, in two groups: younger than 7 years of age or ≥ 7-years-old. In regards to outcome, they were categorized to either ICCP, if they fulfilled the criteria at any time point during follow-up, or simple PT. The patients were aged 6.7-7.3 years at initial evaluation, and 40% of the children diagnosed with ICPP in this study underwent a positive GnRH stimulation test. The other 60% in the ICPP group did not undergo the GnRH stimulation test, whose LH and FSH levels tended to increase progressively with enlargement of the uterus and ovaries during follow-up until 8 years of age. The five diagnostic criteria were simultaneously met for the diagnosis of true precocious puberty. A flowchart of participant screening and enrollment is shown in **Figure 1**.

### Outcomes and timing of diagnosis in girls with early breast development

**Figure 2:**
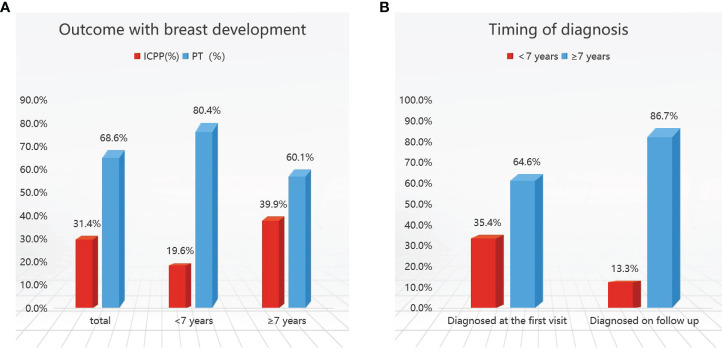
Outcomes **(A)** and timing of diagnosis **(B)** in girls with early breast development. ICPP, idiopathic central precocious puberty; PT, premature thelarche.

Of the 452 girls with follow-up, 142 (31.4%) were finally diagnosed with ICPP; the other 310 (68.6%) were diagnosed with PT. Further stratification revealed that 189 (41.8%) patients were <7 years old, and 263 (58.2%) were ≥7 years old. In the <7-year-old group, 37/189 (19.6%) patients were diagnosed with CPP, compared to 105/263 (39.9%) in the ≥7-year-old group (**Figure 2**).

Girls who met the five diagnostic criteria based on the 2015 Chinese guidelines (1) at initial evaluation at our hospital outpatient center were diagnosed with CPP (82 patients), accounting for 18.1% of the total patients, and followed up for six months to one year. Those who met 2 or 3 diagnostic criteria were followed up until the age of 8 years and were confirmed to have CPP were considered to be diagnosed on follow-up (60 patients), representing 13.3% of the total patients. A total of 142 children were diagnosed with CPP including 82 patients at initial evaluation and 60 patients at follow-up. ICPP was diagnosed less frequently at initial evaluation in those younger than 7 years of age, accounting for 35.4% to 64.6% (**Table 1**).

**Table 1:** Outcomes in girls with early breast development.

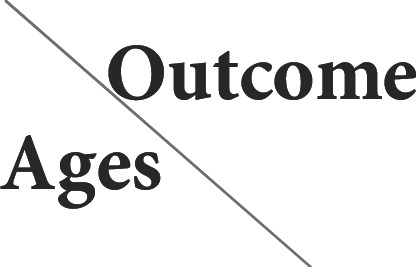	ICPP	Diagnosed at the first visit:	Diagnosed on follow up	PT	Regression	Persistence	Cycle	Progressive
Total (452)	142 (31.4%)	82 (18.1%)	60 (13.3%)	310 (68.6%)	41 (13.2%)	141 (45.4%)	78 (25.2%)	50 (16.1%)
< 7 years (189)	37 (19.6%)	29 (35.4%)	8 (13.3%)	152 (80.4%)	29 (70.7%)	69 (8.9%)	33 (42.3%)	21 (42.0%)
≥ 7 years (263)	105 (39.9%)	53 (64.6%)	52 (86.7%)	158 (60.1%)	12 (29.3%)	72 (51.1%)	45 (57.7%)	29 (58.0%)

ICPP, idiopathic central precocious puberty; PT, premature thelarche.

In girls with PT, during follow-up, 13.2% had breast regression and from those 25.2% presented with recurrence of thelarche during follow-up. No significant progression after breast development was observed in 45.4% of the patients, whereas 16.1% of the patients showed enlargement of glandular tissue which did not progress more than Tanner stage III prior to the age of 8 years (**Table 1**).

The present results suggest that the majority of cases of premature breast development were attributed to PT.

### Comparison of the physical and sexual development between the ICPP and PT groups

**Figure 3:**
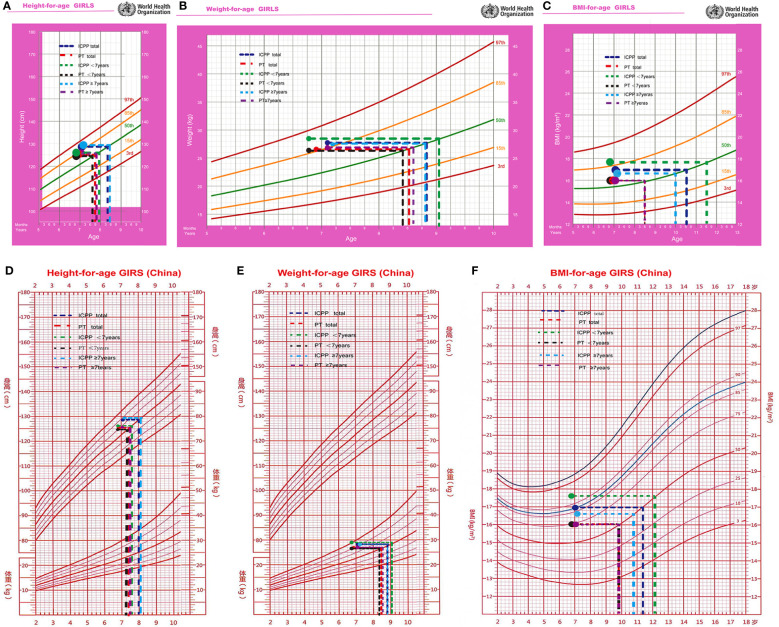
The height **(A, D)**, weight **(B, E)**, and BMI **(C, F)** were equivalent to normal girl's age at initial evaluation.

Compared with that of normally developing girls, the physical development of girls with PT is advanced. The median age in PT group was 6.9 years, with a mean height of 125.3 cm (equivalent to a normally developing girl aged 7.7 years(WHO growth charts, 50th percentile)), a mean weight of 26.5 kg (equivalent to a normally developing girl aged 8.5 years), a mean BMI of 16.0 kg/m^2^ (equivalent to a normally developing girl aged 8.5 years) (**Figure 3**), and a BA of 7.6 years. The comparative analysis of the physical status of the children at initial evaluation in the 2 groups revealed that the ICPP group had significantly higher height, weight, and BMI as compared with the PT group (P < 0.05). Their anthropometrics and laboratory values are listed in **Table 2**.

**Table 2:** Physical and sexual development at the initial visit in the ICPP and PT groups.

	Total			<7 years			≥7 years		
	ICPP (n=142)	PT (n=310)	P value	ICPP (n=37)	PT (n=152)	P value	ICPP (n=105)	PT (n=158)	P value
Chronological age (yr)	7.1 ± 0.38	6.9 ± 0.29	<0.01	6.8 ± 0.17	6.8 ± 0.27	0.721	7.2 ± 0.10	7.1 ± 0.11	<0.01
Height SDS	1.30 ± 1.03	0.84 ± 0.87	0.010	1.46 ± 1.05	1.12 ± 0.85	0.239	1.31 ± 1.07	0.77 ± 0.88	0.011
Height(cm)	128.4 ± 5.70	125.3 ± 5.27	<0.01	126.1 ± 4.86	124.8 ± 4.46	0.185	129.2 ± 5.44	125.7 ± 5.99	<0.01
Age of height equivalent in a normally developing girl (yr) (WHO)	8.3	7.7		7.9	7.6		8.4	7.8	
Age of height equivalent in a normally developing girl (yr) (China)	8	7.4		7.6	7.3		8.1	7.5	
Weight SDS	1.67 ± 1.21	0.94 ± 0.99	<0.01	2.07 ± 1.44	1.26 ± 1.13	0.056	1.46 ± 1.22	0.997 ± 1.15	0.275
Weight (kg)	27.9 ± 4.28	26.5 ± 3.21	<0.01	28.3 ± 4.84	26.3 ± 3.59	0.047	27.8 ± 4.30	26.7 ± 4.90	0.096
Age of weight equivalent in a normally developing girl (yr) (WHO)	8.8	8.5		9.1	8.4		8.8	8.6	
Age of weight equivalent in a normally developing girl (yr) (China)	8.8	8.5		9.1	8.4		8.8	8.6	
BMI SDS	0.79 ± 0.95	0.04 ± 1.79	0.018	1.12 ± 1.14	0.39 ± 2.16	0.162	0.63 ± 0.91	-0.16 ± 1.86	0.028
BMI (kg/m^2)^)	16.9 ± 1.80	16.0 ± 1.50	<0.01	17.6 ± 2.07	16.0 ± 1.81	0.112	16.6 ± 2.0	16.0 ± 2.41	0.711
Age of BMI equivalent in a normally developing girl (yr) (WHO)	10.5	8.5		11.5	8.5		10	8.5	
Age of BMI equivalent in a normally developing girl (yr) (China)	11.3	9.8		12.1	9.8		10.8	9.8	
BA (yr)	9.2 ± 1.01	7.55 ± 1.16	<0.01	8.31 ± 0.75	7.36 ± 1.05	<0.01	9.33 ± 0.98	7.67 ± 1.20	<0.01
Basal LH (IU/L)	0.48 ± 0.53	0.15 ± 0.15	<0.01	0.50 ± 0.64	0.16 ± 0.16	<0.01	0.47 ± 0.46	0.14 ± 0.14	<0.01
Basal FSH (IU/L)	3.62 ± 2.24	2.35 ± 1.23	<0.01	3.52 ± 1.80	2.29 ± 1.16	<0.01	3.67 ± 2.45	2.48 ± 1.35	<0.01
E2 (pg/ml)	33.5 ± 19.3	26.1 ± 15.8	<0.01	31.5 ± 20.3	27.6 ± 16.1	0.208	34.6 ± 18.8	24.8 ± 15.6	<0.01
Uterine diameter (cm)	3.85 ± 0.57	3.49 ± 0.52	<0.01	3.71 ± 0.62	3.52 ± 0.49	0.079	3.92 ± 0.53	3.46 ± 0.56	<0.01
Ovarian volume (ml)	1.64 ± 0.52	1.36 ± 0.46	<0.01	1.55 ± 0.47	1.32 ± 0.46	0.022	1.68 ± 0.54	1.38 ± 0.46	<0.01
Number of follicles (>4mm)	3.79 ± 2.11	2.93 ± 1.89	<0.01	3.61 ± 1.62	3.23 ± 2.13	0.364	3.86 ± 2.28	2.63 ± 1.58	<0.01

ICPP, idiopathic central precocious puberty; PT, premature thelarche; SDS, standard deviation score; BMI, body mass index; WHO, World Health Organization; BA, bone age; LH, luteinizing hormone; FSH, follicle stimulating hormone; E2, estrogen.

In ICPP group, the children’s median age at initial evaluation was 6.9 years, with a mean height of 125.6 cm (equivalent to a normally developing girl aged 7.7 years), a mean weight of 25.3 kg (equivalent to a normally developing girl aged 8.2 years), a mean BMI of 16.1 kg/m^2^ (equivalent to a normally developing girl aged 8.6 years), and a BA of 7.6 years. The median age of the children at the time of ICPP diagnosis was 7.3 years, with a minimum age of 6.7 years and a maximum age of 8 years; their mean height was 129.6 cm (equivalent to a normally developing girl aged 8.5 years), their mean weight was 28.4 kg (equivalent to a normally developing girl aged 9.1 years), their mean BMI was 17.0 kg/m^2^ (equivalent to a normally developing girl aged 10.5 years), and their mean BA was 9.2 years (**Table 3**).

**Table 3:** Physical and sexual development at the initial visit and the time of CPP diagnosis in girls with early breast development.

	At initial visit (n=370)	Time of ICPP diagnosis (n=142)	P value
Chronological age (yr)	6.9 ± 0.24	7.3 ± 0.38	<0.01
Height (cm)	125.6 ± 5.33	129.6 ± 5.79	<0.01
Age of height equivalent in a normally developing girl (yr) (WHO)	7.7	8.5	
Age of height equivalent in a normally developing girl (yr) (China)	7.4	8.2	
Weight (kg)	25.3 ± 3.37	28.4 ± 4.48	<0.01
Age of weight equivalent in a normally developing girl (yr) (WHO)	8.2	9.1	
Age of weight equivalent in a normally developing girl (yr) (China)	8.2	9.1	
BMI (kg/m^2)^)	16.1 ± 1.49	17.0 ± 1.81	<0.01
Age of BMI equivalent in a normally developing girl (yr) (WHO)	8.6	10.5	
Age of BMI equivalent in a normally developing girl (yr) (China)	10	11.4	
BA (yr)	7.55 ± 1.16	9.2 ± 1.01	<0.01
LH (IU/L)	0.18 ± 0.30	0.87 ± 1.12	<0.01
FSH (IU/L)	2.48 ± 1.32	4.11 ± 2.53	<0.01
E2 (pg/ml)	26.1 ± 15.8	37.8 ± 19.5	<0.01
Uterine diameter (cm)	3.41 ± 0.41	3.99 ± 0.54	<0.01
Ovarian volume (ml)	1.80 ± 0.59	1.38 ± 0.46	<0.01
Number of follicles (>4mm)	4.5 ± 2.01	2.85 ± 1.82	<0.01

ICPP, idiopathic central precocious puberty; BMI, body mass index; WHO, World Health Organization; BA, bone age; LH, luteinizing hormone; FSH, follicle stimulating hormone; E2, estrogen.

### Significant parameters found between the CPP and PT girls that can predict the outcome of early breast development

The physical and sexual development parameters considered to be related to the outcome of early breast development were assessed. Logistic regression analysis was carried out to identify the variables affecting the diagnosis of precocious puberty. BMI (odds ratio [OR], 1.37; 95% confidence interval [CI], 1.020–1.842; *P*<0.005), basal LH level (OR, 1.19; 95% CI, 1.021–1.382; *P*<0.005), and uterine diameter (OR, 2.65; 95% CI: 0.982-7.143; *P*=0.05) were significantly related to the diagnosis of precocious puberty, whereas basal E2 level and ovarian volume were not (**Table 4**). Therefore, BMI, basal LH level and uterine diameter were considered predictors of the diagnosis of CPP.

**Table 4:** Binary logistic regression analysis of the parameters related to CPP.

Variable	Odds ratio	95% CI	P value
BMI (kg/m^2^)	1.37	1.020-1.842	0.036
Basal LH (IU/L)	1.19	1.021-1.382	0.026
E2 (pg/ml)	1.10	0.953-1.272	0.19
Uterine diameter (cm)	2.65	0.982-7.143	0.050
Ovarian volume (ml)	1.91	0.570-6.362	0.295

CPP, central precocious puberty; BMI, body mass index; LH, luteinizing hormone; E2, estrogen.

**Figure 4:**
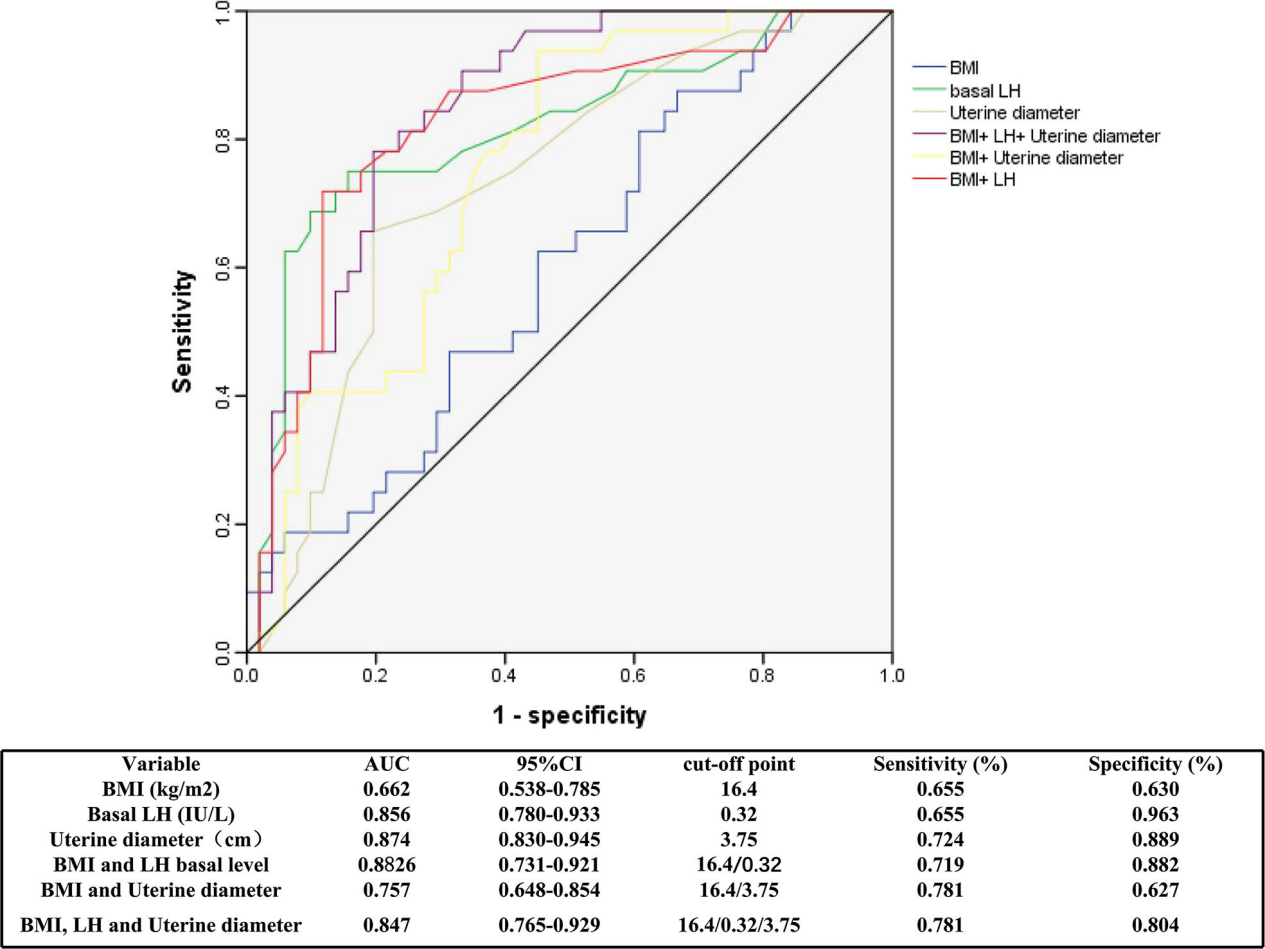
Receiver operator characteristic curves of BMI, basal LH and uterine diameter for the diagnosis of CPP. ROC, receiver operating characteristic; AUC, area under the curve; BMI, body mass index; LH, luteinizing hormone; CPP, central precocious puberty.

The optimal cutoff values for BMI, LH level and uterine diameter to discriminate between girls with CPP and PT were determined using ROC curves (**Figure 4**), which were based on logistic regression analyses. The AUCs for BMI, basal LH level and uterine diameter were 0.766, 0.727, and 0.769, respectively. The optimal cutoff value for each parameter was selected using the Youden index (J) based on ROC analyses. The cutoff value for BMI was 16.4 kg/m^2^, with a sensitivity of 65.5% and a specificity of 63% (AUC, 0.662). When the basal LH level or uterine diameter was combined with BMI to predict CPP, the specificity and sensitivity were more satisfactory. The sensitivity and specificity for each cutoff value are shown in **Figure 4**.

## Discussion

In this study, a total of 452 girls who presented with early breast development between 6.7 and 7.2 years of age were assessed; 31.4% were ultimately diagnosed with CPP, and 68.6% were diagnosed with PT. Previous studies reported that the percentage of girls with early breast development progressing to precocious puberty varied from 9% - 38% (13–15). If the onset age of breast development is older (especially over 2 years), there is more correlation with precocious puberty (16–18). The percentage of girls with early breast development who developed precocious puberty in present study was higher than that previously reported in the literature, possibly attributed to the older age of this study group. In present study, 19.6% of the children younger than 7 years of age, and 39.9% older than 7 years of age developed CPP. Current data also indicates that the probability of CPP is lower in the younger age group than in the higher age group.

The majority of the girls with early breast development in present study were diagnosed with PT. This demonstrates that atypical isolated breast development is common in older girls. Multiple studies have shown that breast development before 2 years of age does not progress or retract in 89-96% of patients, whereas children after 2 years of age with breast development are more likely to exhibit progression, and 11-13% progress to CPP (19, 20). The present study similarly found that the rate of breast regression or nonprogression was significantly higher in the younger group. In CPP, the gonads develops earlier, but breast development is not always accompanied by ovarian development. PT does not necessarily lead to progressive ovarian development. Present study verified guidelines stating that breast development does not equate to CPP (21, 22). Therefore, follow-up observation of these patients is very important.

Several studies and epidemiological reports have noted that obesity in girls is a risk factor for early pubertal development (23–26). Research in females suggests that obesity is more likely to lead to precocious puberty (27, 28). In present study, The physical development, including height, weight and BMI measurements, of girls with early breast development, compared with that of normally developing girls, was significantly advanced corresponding to the mean values for girls older by 1 year, finding consistent with reports of other studies (28–32). Girls in the CPP group had higher weight, height, and BMI measurements than those in the PT group. When ICPP was diagnosed, the mean BMI of the girls’ physical development reached 17.0 kg/m^2^, corresponding to that of a 10.5-year-old girl. Therefore, for obese children, their sexual development progression should be more closely monitored. Educating their families to control weight and to adopt healthy lifestyles to prevent precocious puberty is necessary. In addition, it was proven that the early development of the breast was also preceded by physical development. Therefore, there is no need for excessive anxiety in children with early sexual development, because their physical development is similarly advanced. However, prompt recognition and treatment are necessary in children with rapid sexual development progression.

In this study cohort, compared to those with PT, girls with CPP had an advanced BA at diagnosis. The levels of LH, FSH, and E2, in girls with precocious puberty may not be elevated at initial evaluation but may gradually rise and become accompanied by enlargement of the uterus and ovaries during follow-up. However, there was no significant progression or slow progression of the breasts in girls with PT, and some girls with breast development with appropriate for age BA and hormonal levels, exhibited spontaneous breast regression. Therefore, it is clinically important to closely monitor changes in breast development, BA, hormone levels, and the size of uterus and ovaries in girls with early breast development. However, advanced BA does not always signifies precocious puberty, as it can be also attributed to peripheral hormonal conversion in the adipose tissue of girls with obesity.

To date, there is an increasing emphasis on basal sex hormone levels for diagnosing CPP because of the insurmountable drawbacks of the nonphysiological GnRH stimulation test, which does not truly reflect the level of gonadal development and requires multiple blood samples, so clinicians have searched for more reliable, feasible and convenient indicators to diagnose CPP. Pelvic ultrasound indices overlap more in children with PT and precocious puberty and therefore do not have high value for predicting CPP (33). It has been mentioned in the literature that basal gonadotropin levels are useful, but different studies have different cutoff values for diagnosing CPP, so the ideal cutoff value for diagnosing CPP is difficult to determine (34–36). Given that the GnRH stimulation test is cumbersome to perform and that overdiagnosis of CPP due to false positives after the GnRH stimulation test has been documented in the infant population (16), we focused on physical signs and a combination of biomarkers for diagnosing CPP.

In present study, Children with ICPP had significantly higher BMIs and BMI standard deviation scores (SDSs) than children with PT. We further found by ROC curve analysis that at a BMI cutoff point of 16.4 kg/m^2^ for diagnosing CPP, the sensitivity was 65.5%, and the specificity was 63%. In addition, BMI combined with other indicators, such as a uterine diameter of 3.75 cm and a basal LH value of 0.32 IU/L, exhibited improved sensitivity and specificity. BMI is a stable and macroscopic indicator, and when a girl’s BMI reaches that of a normally developing 10.5-year-old, it predicts the initiation of puberty. In addition to the five classic diagnostic criteria, CPP is diagnosed when physical development corresponds to the mean of 10.5 years old girls. Although physical development is also advanced in girls with PT compared with normal girls, the critical point is reached when CPP occurs. When girls have a markedly elevated BMI, CPP can be diagnosed in combination with clinical presentation, which reduces the need for the GnRH stimulation test. In particular, the association between physical and pubertal development is further confirmed.

In conclusion, a large number of girls with isolated breast development were assessed, including children in an older age group; therefore, clinical follow-up for this subset is important. Girls with premature breast development had earlier physical development than normally developing girls and required vigilant monitoring for the onset of precocious puberty when BMI reached that of a normally developing 10.5-year-old child. BMI is an independent risk factor for early pubertal development. Therefore, girls who are overweight or obese should be encouraged to control weight, and the progress of sexual development should be more closely followed. This study addresses the cutoff values for physical development and BMI measurements and demonstrates that the initiation of pubertal development, manifested by the attainment of a boundary value for body mass, is preceded by the attainment of physical and sexual organ development, skeletal maturation, and elevated hormone levels. The intrinsic implication of puberty was extended to be that initiation of pubertal development is not only a trigger for factors but also an extrinsic manifestation of multiple intrinsic alterations of factors. BMI is a simple and clear predictor of ICPP. In addition to the five classic diagnostic criteria, CPP is diagnosed when physical development corresponds to that of a 10.5 year old.

This study has several limitations. The study design was retrospective, and the girls in this study are currently in the process of sexual development. We will conduct multicenter studies and further prospective studies to observe the physical appearance of these patients when they reach a final height in adulthood.

## Data availability statement

The original contributions presented in the study are included in the article/supplementary material. Further inquiries can be directed to the corresponding author.

## Ethics statement

The studies involving human participants were reviewed and approved by Written informed consent was obtained from a parent or guardian on behalf of each child and the study was approved by the ethics committee of Beijing Children’s Hospital, Capital Medical University. Written informed consent to participate in this study was provided by the participants’ legal guardian/next of kin.

## Author contributions

MZ collected clinical samples and wrote the manuscript; ML and BC contributed to the project management; CG conceived and designed the project and revised the manuscript. All authors contributed to the article and approved the submitted version.

## Acknowledgments

The study was supported by the patients and the cooperation of their families and the help of doctors and nurses in the Department of Endocrinology.

## Conflict of interest

The authors declare that the research was conducted in the absence of any commercial or financial relationships that could be construed as a potential conflict of interest.

## Publisher’s note

All claims expressed in this article are solely those of the authors and do not necessarily represent those of their affiliated organizations, or those of the publisher, the editors and the reviewers. Any product that may be evaluated in this article, or claim that may be made by its manufacturer, is not guaranteed or endorsed by the publisher.
